# Endocrine cell type sorting and mature architecture in the islets of Langerhans require expression of Roundabout receptors in β cells

**DOI:** 10.1038/s41598-018-29118-x

**Published:** 2018-07-18

**Authors:** Melissa T. Adams, Jennifer M. Gilbert, Jesus Hinojosa Paiz, Faith M. Bowman, Barak Blum

**Affiliations:** Department of Cell and Regenerative Biology, University of Wisconsin-Madison School of Medicine and Public Health, 1111 Highland Ave., Madison, WI 53705 USA

## Abstract

Pancreatic islets of Langerhans display characteristic spatial architecture of their endocrine cell types. This architecture is critical for cell-cell communication and coordinated hormone secretion. Islet architecture is disrupted in type-2 diabetes. Moreover, the generation of architecturally correct islets *in vitro* remains a challenge in regenerative approaches to type-1 diabetes. Although the characteristic islet architecture is well documented, the mechanisms controlling its formation remain obscure. Here, we report that correct endocrine cell type sorting and the formation of mature islet architecture require the expression of Roundabout (Robo) receptors in β cells. Mice with whole-body deletion of *Robo1* and conditional deletion of *Robo2* either in all endocrine cells or selectively in β cells show complete loss of endocrine cell type sorting, highlighting the importance of β cells as the primary organizer of islet architecture. Conditional deletion of *Robo* in mature β cells subsequent to islet formation results in a similar phenotype. Finally, we provide evidence to suggest that the loss of islet architecture in *Robo KO* mice is not due to β cell transdifferentiation, cell death or loss of β cell differentiation or maturation.

## Introduction

The islets of Langerhans display typical, species-specific architecture, with distinct spatial organization of their various endocrine cell types^1–5^. In the mouse, the core of the islet is composed mostly of insulin-secreting β cells, while glucagon-secreting α cells, somatostatin-secreting δ cells and pancreatic polypeptide-secreting PP cells are located at the islet periphery^3^. In humans and other primates, islet architecture is more complex, but still conforms to the overall structure of several β cell lobules surrounded by mantles of α, δ and other endocrine cells types^4,5^. Correct islet architecture facilitates the mature pattern of hormone release, directionality of intra-islet paracrine signaling, and connection with the microvasculature^6,7^. The typical islet architecture is disrupted in obesity, insulin resistance, and diabetes in both humans and rodents^8–14^. Structural islet integrity and architecture are also disrupted in cadaver islets during isolation and culture prior to islet transplantation, as well as after infusion into the portal vein^15–18^. Moreover, the generation of *bona fide* islets of Langerhans from human pluripotent stem cells, in which the three-dimensional islet architecture is recapitulated, remains a pressing challenge in regenerative medicine approaches to diabetes^19,20^.

The formation of the islets of Langerhans in the mouse starts with the delamination of individual pro-endocrine cells from the pancreatic duct, beginning at embryonic day (E) 13.5^21^. These cells then migrate into the mesenchyme, aggregate to form proto-islet clusters, and subsequently rearrange into the typical mantle/core architecture of the mature islets of Langerhans^22^. Interestingly, dissociated rat islets re-aggregate spontaneously in culture, recapitulating the original mantle-core islet architecture, suggesting that the signals and forces controlling islet architecture are islet-autonomous^23^. Despite the four decades that have passed since the typical islet architecture was first described^24,25^, the mechanisms controlling the formation of mature islet architecture during development and its maintenance in the adult remain largely unresolved^22,26^.

Roundabout (Robo) receptors are cell surface receptors that bind the ligand Slit, originally recognized for their involvement in axon guidance and neuronal migration^27^. Among the four Robo family members, Robo1 and Robo2 were shown to be expressed in the islets of Langerhans of both humans and rodents^28–33^. Furthermore, *in vitro* analyses illustrate that Slit-Robo signaling in the islet can improve β cell survival during stress and hyperglycemia and to potentiate insulin secretion^33^. However, the role of this pathway in the islet *in vivo* has not yet been demonstrated.

It recently was shown that a double deletion of *Robo1* and *Robo2* in lung pulmonary neuroendocrine cells (PNECs) results in the loss of PNECs’ clustered architecture^34^. The delamination, migration and aggregation of the islets of Langerhans involve several Robo-related neuronal proteins such as Semaphorin, Ephrin/Eph and N-CAM^35–40^, as well as direct signals from the nervous system^41^. Moreover, Robo receptors themselves have been implicated in collective cell movement during organogenesis in various mammalian tissues^42,43^. We thus hypothesized that beyond their role in insulin secretion and β cell survival, Robo receptors may also be involved in the organogenesis of the islets of Langerhans, similar to their role in PNECs in the lung.

Here, we show that expression of Robo receptors in β cells is required for endocrine cell type sorting and mature islet architecture. Mice lacking *Robo1* and *Robo2* in all endocrine cells or selectively in β cells show complete loss of endocrine cell type sorting in the islets. Moreover, deletion of Robo receptors in mature β cells after islet formation has been completed also results in intermixing of endocrine cell types and loss of islet architecture. Finally, lineage-tracing experiments in β cell-selective *Robo* knockouts (*Robo KO*) provide evidence suggesting that disruption of islet architecture in *Robo KO* mice is not due to transdifferentiation, β cell death, or insufficient β cell differentiation or maturation.

## Results

### Robo receptors are required for endocrine cell type sorting and mature architecture of the islets of Langerhans

Current understanding of the formation of the mature architecture of the islets of Langerhans during development suggests that, beginning at E13.5, individual endocrine progenitors within the pancreatic duct independently turn on the transcription factor Neurogenin3 (Neurog3), and delaminate from the duct into the surrounding mesenchyme as single cells. These delaminated cells then migrate away from the duct and coalesce to form the mature islet architecture^35,37,44,45^. To test our hypothesis that Robo receptors are involved in the organogenesis of the islets of Langerhans, we generated an early endocrine progenitor knockout of *Robo* by crossing *Robo1*^*Δ/Δ*^,*2*^*flx/flx*^ mice^34^ with *Neurog3-Cre* mice^46^. *Robo1*^*Δ/Δ*^,*2*^*flx/flx*^ mice harbor a linked *Robo1* deletion allele (*Robo1*^*Δ*^) and conditional *Robo2* deletion allele (*Robo2*^*flx*^). Neurog3 is expressed in all endocrine progenitors during their delamination from the duct, and its expression is subsequently turned off prior to endocrine cell aggregation to form proto-islet clusters^47,48^. The resulting *Neurog3-Cre;Robo1*^*Δ/Δ*^,*2*^*flx/flx*^ mice carry a whole-body deletion of *Robo1*, and a pancreatic endocrine-selective deletion of *Robo2*. This strategy was chosen to eliminate redundant Robo signaling and to avoid the homozygous lethality of *Robo2* whole-body deletion^49^. *Neurog3-Cre;Robo1*^*Δ/Δ*^,*2*^*flx/flx*^ progeny are viable and appear normal.

**Figure 1:**
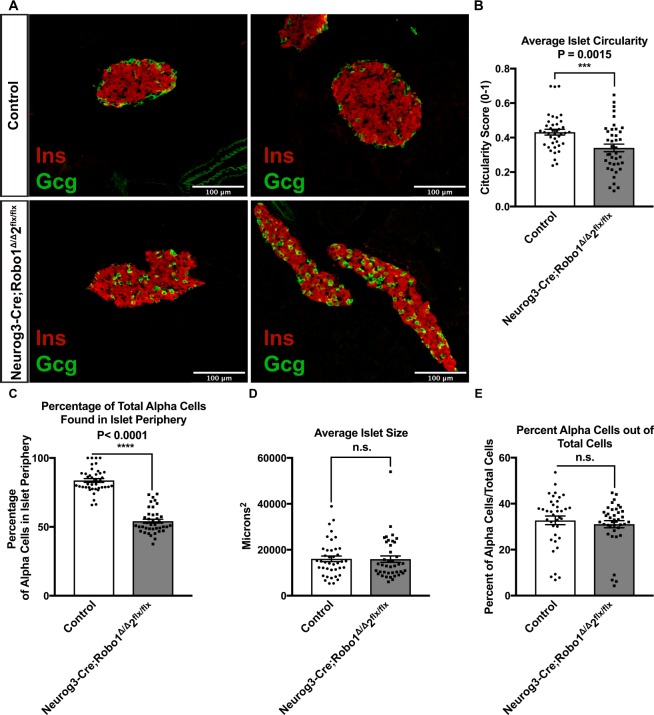
Robo receptors are required for endocrine cell type sorting and mature architecture of the islets of Langerhans. **(A)** Immunofluorescence staining for β cells (Insulin, red) and α cells (glucagon, green) of control (*Robo1*^+*/Δ*^,*2*^+*/flx*^ and *Neurog3-Cre;Robo1*^+*/*+^,*2*^+*/*+^) and *Neurog3-Cre;Robo1*^*Δ/Δ*^,*2*^*flx/flx*^ islets from 2 month old mice. **(B)** Average Circularity Index of control (*Robo1*^+*/Δ*^,*2*^+*/flx*^ and *Neurog3-Cre;Robo1*^+*/*+^,*2*^+*/*+^) and *Neurog3-Cre;Robo1*^*Δ/Δ*^,*2*^*flx/flx*^ islets. **(C)** Percentage of total α cells found in periphery of the islet in control (*Robo1*^+*/Δ*^,*2*^+*/flx*^ and *Neurog3-Cre;Robo1*^+*/*+^,*2*^+*/*+^) *vs*. *Neurog3-Cre;Robo1*^*Δ/Δ*^,*2*^*flx/flx*^ mice. **(D)** Average size of control (*Robo1*^+*/Δ*^,*2*^+*/flx*^ and *Neurog3-Cre;Robo1*^+*/*+^,*2*^+*/*+^) and *Neurog3-Cre;Robo1*^*Δ/Δ*^,*2*^*flx/flx*^. **(E)** Percent of α cells out of total cells in control (*Robo1*^+*/Δ*^,*2*^+*/flx*^ and *Neurog3-Cre;Robo1*^+*/*+^,*2*^+*/*+^) and *Neurog3-Cre;Robo1*^*Δ/Δ*^,*2*^*flx/flx*^.

Upon histological analysis of pancreata from adult (2 months old) *Neurog3-Cre;Robo1*^*Δ/Δ*^,*2*^*flx/flx*^ mice we found that proto-islets do form in the absence of Robo signaling in endocrine cells (Fig. 1A), suggesting that Robo is not required for endocrine cell aggregation. Strikingly, most *Neurog3-Cre;Robo1*^*Δ/Δ*^,*2*^*flx/flx*^ islets are long, thin, disorganized, and lie directly along the duct (Fig. 1A, lower right panel). Consistent with their lengthy appearance, the Circularity Index of *Neurog3-Cre;Robo1*^*Δ/Δ*^,*2*^*flx/flx*^ islets is significantly decreased compared to controls (Circularity Index = 0.43 ± 0.02 for controls and 0.33 ± 0.02 for *Neurog3-Cre;Robo1*^*Δ/Δ*^,*2*^*flx/flx*^, *P* = 0.0015, *n* = 10–20 islets each for two independent mice in each genotype) (Fig. 1B). Moreover, both α and δ cells in islets of *Neurog3-Cre;Robo1*^*Δ/Δ*^,*2*^*flx/flx*^ mice lose their stereotypical location in the islet periphery (the two outermost layers of cells in the islet), and instead are intermingled with β cells throughout the islet. Thus, while in control mice, 84% and 81% of α and δ cells, respectively, are located in the islet periphery, only 39% and 54% of α and δ cells, respectively, are located in the islet periphery in islets of *Neurog3-Cre;Robo1*^*Δ/Δ*^,*2*^*flx/flx*^ mice (*P* < 0.0001, *n* = 10–20 islets each for two independent mice in each genotype) (Fig. 1C, Supplemental Fig. 2A,B). The overall islet size, on the other hand, was similar between *Neurog3-Cre;Robo1*^*Δ/Δ*^,*2*^*flx/flx*^ and controls (15918 µm^2^ and 16058 µm^2^, respectively) (Fig. 1D). Furthermore, there was no significant difference in α cells as a percentage of total islet cells between control and *Neurog3-Cre;Robo1*^*Δ/Δ*^,*2*^*flx/flx*^ mice (Fig. 1E). Islets of control *Robo1*^*Δ/Δ*^,*2*^*flx/flx*^ and *Robo1*^+*/Δ*^,*2*^+*/flx*^ mice without the *Cre* transgene show normal islet architecture, indicating that loss of *Robo1* alone is not sufficient for the observed disruption of islet architecture (Supplemental Fig. 1).

### Deletion of Robo receptors in β cells alone is sufficient to disrupt endocrine cell type sorting and islet architecture

Because deletion of Robo receptors in *Neurog3-Cre;Robo1*^*Δ/Δ*^,*2*^*flx/flx*^ mice results in disruption of islet architecture, without apparent interference with endocrine cell aggregation into proto-islet clusters, we hypothesized that the deletion of *Robo* may affect later events in islet organogenesis. To test this hypothesis, we generated a β cell-selective deletion of *Robo* by crossing *Robo1*^*Δ/Δ*^,*2*^*flx/flx*^ mice with an *Insulin-Cre* line (*Ins2-Cre*)^50^.

**Figure 2:**
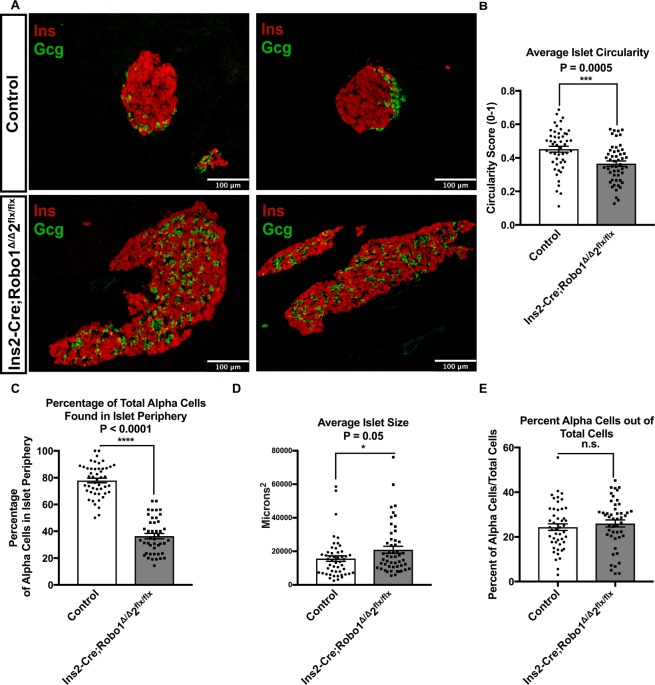
Deletion of Robo receptors in β cells alone is sufficient to disrupt endocrine cell type sorting and islet architecture. **(A)** Immunofluorescence staining for β cells (Insulin, red) and α cells (glucagon, green) of control (*Robo1*^+*/*+^,*2*^+*/*+^ and *Ins2-Cre;Robo1*^+*/*+^,*2*^+*/*+^) and *Ins2-Cre;Robo1*^*Δ/Δ*^,*2*^*flx/flx*^ islets from 2 month old mice. **(B)** Average Circularity Index of control (*Robo1*^+*/*+^,*2*^+*/*+^ and *Ins2-Cre;Robo1*^+*/*+^,*2*^+*/*+^) and *Ins2-Cre;Robo1*^*Δ/Δ*^,*2*^*flx/flx*^ islets. **(C)** Percentage of total α cells found in periphery in control (*Robo1*^+*/*+^,*2*^+*/*+^ and *Ins2-Cre;Robo1*^+*/*+^,*2*^+*/*+^) *vs*. *Ins2-Cre;Robo1*^*Δ/Δ*^,*2*^*flx/flx*^ islets. **(D)** Average size of control (*Robo1*^+*/*+^,*2*^+*/*+^ and *Ins2-Cre;Robo1*^+*/*+^,*2*^+*/*+^) and *Ins2-Cre;Robo1*^*Δ/Δ*^,*2*^*flx/flx*^ islets. **(E)** Percent of α cells out of total cells in control (*Robo1*^+*/*+^,*2*^+*/*+^ and *Ins2-Cre;Robo1*^+*/*+^,*2*^+*/*+^) and *Ins2-Cre;Robo1*^*Δ/Δ*^,*2*^*flx/flx*^.

As in the case of *Neurog3-Cre;Robo1*^*Δ/Δ*^,*2*^*flx/flx*^, islets of Langerhans from *Ins2-Cre;Robo1*^*Δ/Δ*^,*2*^*flx/flx*^ mice show marked defects in islet architecture and endocrine cell type sorting compared to islets from control littermates (Fig. 2A). Islets of *Ins2-Cre;Robo1*^*Δ/Δ*^,*2*^*flx/flx*^ are thus less circular than controls (Circularity Index = 0.37 ± 0.02, and 0.45 ± 0.02 for *Ins2-Cre;Robo1*^*Δ/Δ*^,*2*^*flx/flx*^ and controls, respectively, *P* = 0.0005, *n* = 10–20 islets each for three independent mice in each genotype) (Fig. 2B). Likewise, while in control mice, 78% and 83% of α and δ cells, respectively, are located in the islet periphery, only 36% and 46% of α and δ cells, respectively, are located in the islet periphery in islets of *Ins2-Cre;Robo1*^*Δ/Δ*^,*2*^*flx/flx*^ mice (*P* < 0.0001, *n* = 10–20 islets each for three independent mice in each genotype) (Fig. 2C, Supplemental Fig. 3A,B). The overall islet size was significantly different between *Ins2-Cre;Robo1*^*Δ/Δ*^,*2*^*flx/flx*^ and controls (15695 µm^2^ and 20959 µm^2^, respectively, *P* = 0.05, *n* = 10–20 islets each for three independent mice in each genotype) (Fig. 2D). No significant difference in α cells over total islet cells between control and *Ins2-Cre;Robo1*^*Δ/Δ*^,*2*^*flx/flx*^ mice was observed (Fig. 2E).

### Robo receptors are required for endocrine cell type sorting and islet architecture postnatally, and their expression is diminished in obesity

**Figure 3:**
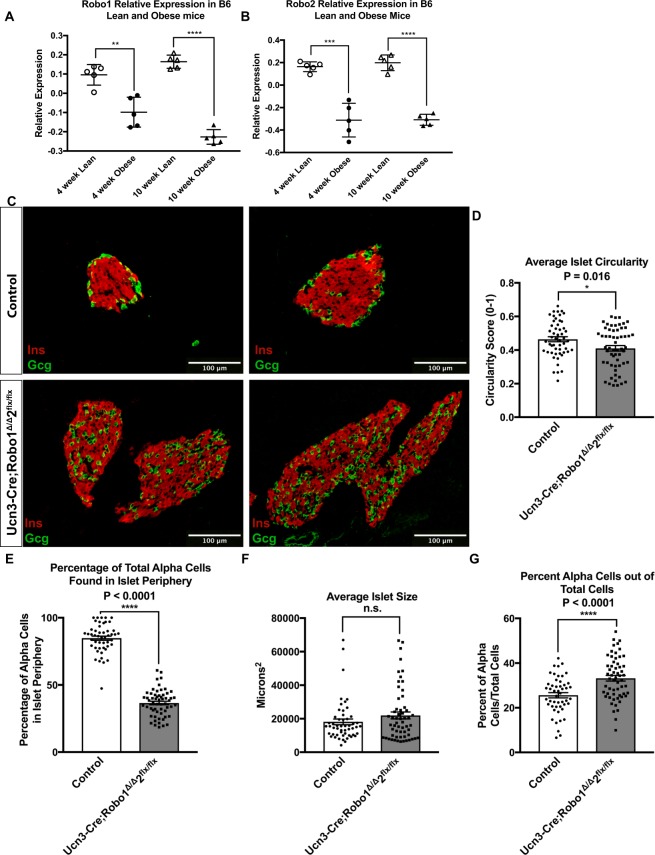
Robo receptors maintain endocrine cell type sorting and islet architecture in the adult, and their expression is diminished in obesity. **(A,B)** Microarray data adapted from Keller and colleagues (Keller *et al*.^51^). Relative expression of *Robo1* **(A)** and *Robo2* **(B)** in lean and obese C57BL6 mice at 4 and 10 weeks. Relative expression is defined here as the log_10_ of the ratio of **(C)** Immunofluorescence staining for β cells (Insulin, red) and α cells (glucagon, green) of control (*Robo1*^+*/*+^,*2*^+*/*+^ and *Ucn3-Cre;Robo1*^+*/*+^,*2*^+*/*+^) and *Ucn3-Cre;Robo1*^*Δ/Δ*^,*2*^*flx/flx*^ islets from 2 month old mice. **(D)** Average Circularity Index of control (*Robo1*^+*/*+^,*2*^+*/*+^ and *Ucn3-Cre;Robo1*^+*/*+^,*2*^+*/*+^) and *Ucn3-Cre;Robo1*^*Δ/Δ*^,*2*^*flx/flx*^ islets. **(E)** Percentage of peripheral α cells in control (*Robo1*^+*/*+^,*2*^+*/*+^ and *Ucn3-Cre;Robo1*^+*/*+^,*2*^+*/*+^) *vs*. *Ucn3-Cre;Robo1*^*Δ/Δ*^,*2*^*flx/flx*^ islets. **(F)** Average size of control (*Robo1*^+*/*+^,*2*^+*/*+^ and *Ucn3-Cre;Robo1*^+*/*+^,*2*^+*/*+^) and *Ucn3-Cre;Robo1*^*Δ/Δ*^,*2*^*flx/flx*^ islets. **(G)** Percent of α cells out of total cells in control (*Robo1*^+*/*+^,*2*^+*/*+^ and *Ucn3-Cre;Robo1*^+*/*+^,*2*^+*/*+^) and *Ucn3-Cre;Robo1*^*Δ/Δ*^,*2*^*flx/flx*^ islets from 2 month old mice.

In some models of type-2 diabetes, islet architecture is distorted, with intermingling of α and β cells reminiscent of the *Neurog3-Cre;Robo1*^*Δ/Δ*^,*2*^*flx/flx*^ and *Ins2-Cre;Robo1*^*Δ/Δ*^,*2*^*flx/flx*^ phenotypes^12^. We therefore wondered whether Robo receptors may be involved in the loss of islet architecture in obesity and diabetes. To test for this possibility, we queried the Genomic Study of Parental Mice database developed by Keller and colleagues^51^, for the expression of *Robo1* and *Robo2*. This database allows searching for gene expression in microarray experiments performed on islets from lean and obese (*Lep*^*ob/ob*^) mice. We found that both *Robo1* and *Robo2* mRNAs are significantly downregulated in islets from obese mice compared to the lean controls as early as 4 weeks of age (Fig. 3A,B). Further literature search revealed that *ROBO1* and *ROBO2* are similarly downregulated in islets from human type-2 diabetics^28,29^. The diminished expression of Robo receptors in islets from obese, pre-diabetic mice and human type-2 diabetics suggests that these genes may be involved in maintaining islet architecture in the adult.

To further test the hypothesis that Robo receptors are important for islet architecture subsequent to initial islet formation, we generated mice with selective deletion of *Robo* in mature β cells. To this end, we crossed *Robo1*^*Δ/Δ*^,*2*^*flx/flx*^ mice with *Urocortin3 (Ucn3)-Cre* mice^52^. Ucn3 is a β cell-selective maturation marker in mice that is highly expressed in β cells starting around postnatal day 10 (P10), subsequent to islet organogenesis^19,53^. Thus, in β cells from *Ucn3-Cre;Robo1*^*Δ/Δ*^,*2*^*flx/flx*^ mice, deletion of *Robo* will occur only after islet architecture has been established. We hypothesized that if Robo signaling is required for islet architecture subsequent to initial islet formation, then the islets in *Ucn3-Cre;Robo1*^*Δ/Δ*^,*2*^*flx/flx*^ mice will have a similar architectural phenotype as those of the *Neurog3-Cre;Robo1*^*Δ/Δ*^,*2*^*flx/flx*^ and *Ins2-Cre;Robo1*^*Δ/Δ*^,*2*^*flx/flx*^ models. However, if Robo receptors are only needed for the initial establishment of islet architecture, then islet architecture in *Ucn3-Cre;Robo1*^*Δ/Δ*^,*2*^*flx/flx*^ mice will not differ from that of the controls. We found that the average circularity index of *Ucn3-Cre;Robo1*^*Δ/Δ*^,*2*^*flx/flx*^ was significantly lower than controls (Circularity Index = 0.41 ± 0.02, and 0.47 ± 0.02 for *Ucn3-Cre;Robo1*^*Δ/Δ*^,*2*^*flx/flx*^ and controls, respectively, *P* = 0.0164, *n* = 10–20 islets each for four independent mice in each genotype) (Fig. 3D). The islets of *Ucn3-Cre;Robo1*^*Δ/Δ*^,*2*^*flx/flx*^ mice still show significant intermingling of α and β cells, similar to that of *Neurog3-Cre;Robo1*^*Δ/Δ*^,*2*^*flx/flx*^ and *Ins2-Cre;Robo1*^*Δ/Δ*^,*2*^*flx/flx*^ mice (Fig. 3C). Thus, while in control mice, 85% of α cells are located in the islet periphery, only 37% of α cells are located in the islet periphery in islets of *Ucn3-Cre;Robo1*^*Δ/Δ*^,*2*^*flx/flx*^ mice (*P* < 0.0001, *n* = 10–20 islets each for four independent mice in each genotype) (Fig. 3E). δ cells behave the same, but were not quantified (Supplemental Fig. 4). The average islet size of *Ucn3-Cre;Robo1*^*Δ/Δ*^,*2*^*flx/flx*^ shows a slight trend towards larger islet than those of controls, but this trend does not reach statistical significance (18157 µm^2^ and 21968 µm^2^, respectively, *P* = 0.1653, *n* = 10–20 islets each for four independent mice in each genotype) (Fig. 3F). Interestingly, we also observed a significant increase in α cell ratio in *Ucn3-Cre;Robo1*^*Δ/Δ*^,*2*^*flx/flx*^ *islets* compared to controls (Fig. 3G).

### The disrupted islet architecture and increase in α cell ratio in *Ucn3-Cre;Robo1*^*Δ/Δ*^,*2*^*flx/flx*^ islets is not due to β cell death, transdifferentiation or defects in differentiation or maturation

**Figure 4:**
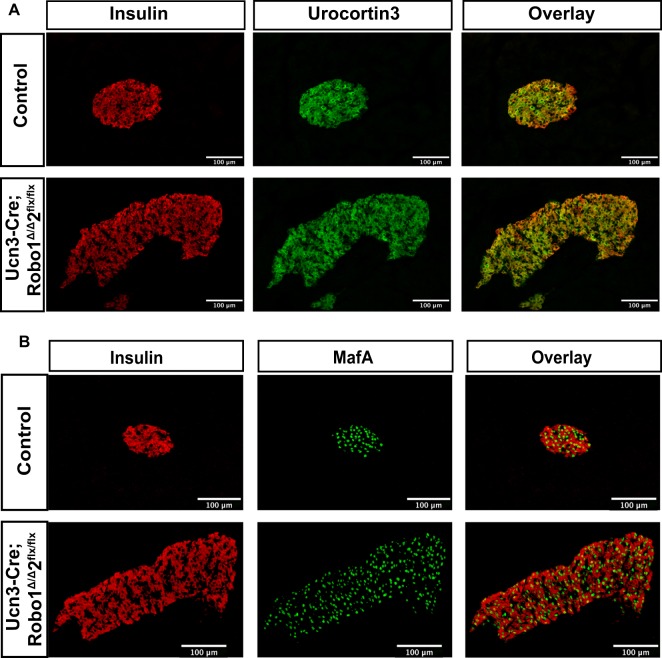
The disrupted islet architecture and increase in α cell ratio in *Robo mβKO* islets is not due to defects in differentiation or maturation of β cells. **(A)** Immunofluorescent staining for Insulin (red) and Ucn3 (green) of control *Ucn3-Cre;Robo1*^+*/*+^,*2*^+*/*+^ and *Ucn3-Cre;Robo1*^*Δ/Δ*^,*2*^*flx/flx*^ islets from 1.5–3 month old mice. **(B)** Immunofluorescent staining for Insulin (red) and MafA (green) of control *Ucn3-Cre;Robo1*^+*/*+^,*2*^+*/*+^ and *Ucn3-Cre;Robo1*^*Δ/Δ*^,*2*^*flx/flx*^ islets from 1.5–3 months old mice. Disorganized islets do not show loss of β cell maturation.

It has been shown that disrupting endocrine cell differentiation and maturation results in disordered islet architecture, reminiscent of our *Robo KO* phenotype^54–61^. To account for this possibility, we compared expression of the β cell maturation markers MafA and Ucn3 in *Ucn3-Cre;Robo1*^*Δ/Δ*^,*2*^*flx/flx*^ mice and controls by immunofluorescence (Fig. 4A,B). Surprisingly, we observed no change in expression of either MafA or Ucn3 in the β cells in *Ucn3-Cre;Robo1*^*Δ/Δ*^,*2*^*flx/flx*^ islets compared to controls, suggesting that deletion of *Robo* results in loss of islet architecture without apparent loss of β cell differentiation and maturation.

**Figure 5:**
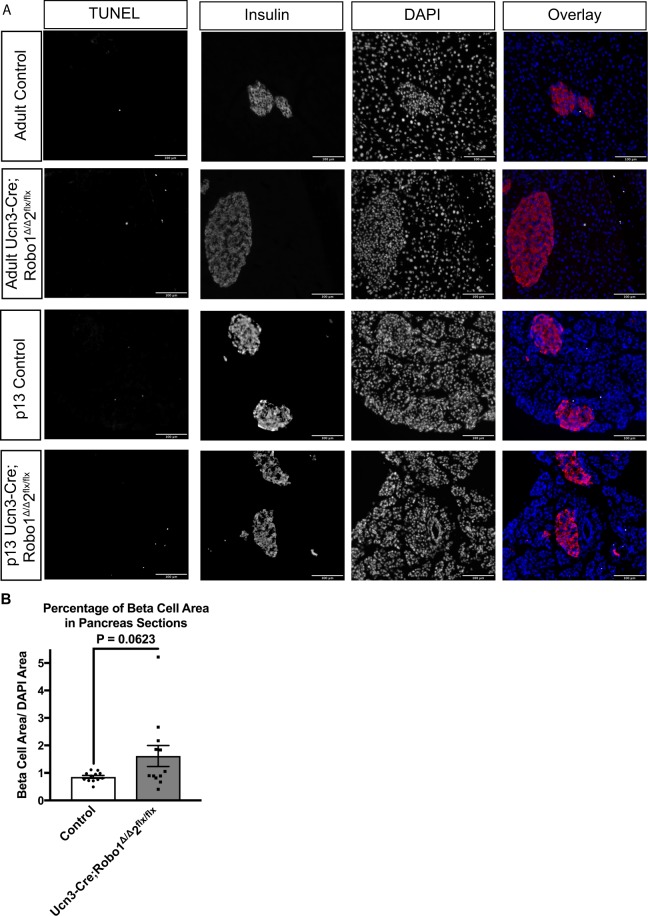
The disrupted islet architecture and increase in α cell ratio in *Ucn3-Cre;Robo1*^*Δ/Δ*^,2^*flx/flx*^ islets is not due to β cell death. **(A)** TUNEL staining for apoptotic cells (white), counterstained with Insulin (red), and DAPI (blue), showing no cell death in adult and p13 control *Ucn3-Cre;Robo1*^+*/*+^,*2*^+*/*+^ and *Ucn3-Cre;Robo1*^*Δ/Δ*^,*2*^*flx/flx*^ islets. **(B)** Percent β cell area of pancreatic sections in control *Ucn3-Cre;Robo1*^+*/*+^,*2*^+*/*+^ and *Ucn3-Cre;Robo1*^*Δ/Δ*^,*2*^*flx/flx*^ showing a trend towards higher β cell area in *Ucn3-Cre;Robo1*^*Δ/Δ*^,*2*^*flx/flx*^.

It has previously been reported that reducing Slit-Robo signaling in islets promotes β cell death, while addition of recombinant Slit ligand improves β cell survival under diabetogenic stresses^33^. This suggests that both the intermixed endocrine cell phenotype and the increase in α cell ratio in *Ucn3-Cre;Robo1*^*Δ/Δ*^,*2*^*flx/flx*^ islets may be caused by β cell death, or replacement of β cells in the islet core by α cells through developmental reprogramming or transdifferentiation. To test the hypothesis that β cell death contributes to these phenotypes, we performed TUNEL analysis on *Ucn3-Cre;Robo1*^*Δ/Δ*^,*2*^*flx/flx*^ islets both at P13-16, when islets are still expanding, and at 8 weeks of age, when islet formation has been completed. We found no increase in α or β cell death in either of these stages (Fig. 5A). Analysis of β cell area in *Ucn3-Cre;Robo1*^*Δ/Δ*^,*2*^*flx/flx*^ islets showed a trend towards larger β cell area in the *Robo KO* when compared to controls though this did not reach statistical significance, further pointing against β cell death in *Robo KO* mice as an explanation for the islet architectural defects (Fig. 5B).

**Figure 6:**
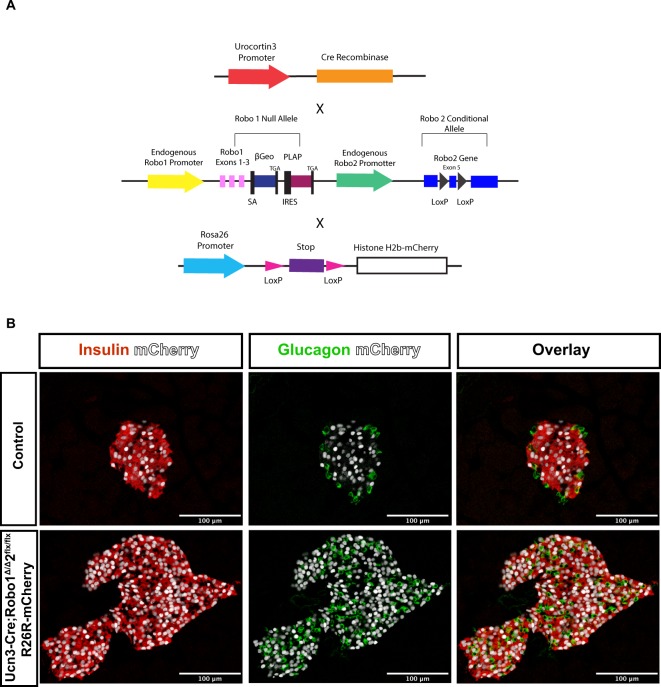
The disrupted islet architecture and increase in α cell ratio in *Ucn3-Cre;Robo1*^*Δ/Δ*^,2^*flx/flx*^ islets is not due to transdifferentiation of β to α cells. **(A)** Schematic of constructs used for lineage tracing *Ucn3-Cre* expressing cells. **(B)** Lineage tracing of mature β cells in control *Ucn3-Cre;Robo1*^+*/*+^,*2*^+*/*+^ and *Ucn3-Cre;Robo1*^*Δ/Δ*^,*2*^*flx/flx*^ islets. Lineage traced β cells express nuclear mCherry (white), and counterstained for Insulin (red), Glucagon (green), and DAPI (blue), showing no transdifferentiation of α to β cells.

To test the hypothesis that β cell transdifferentiation to α cells is the cause of the observed disrupted islet architecture and increase in α cell ratio in *Ucn3-Cre;Robo1*^*Δ/Δ*^,*2*^*flx/flx*^ mice, we performed lineage tracing experiments. To this end, we crossed *Ins2-Cre;Robo1*^*Δ/Δ*^,*2*^*flx/flx*^ or *Ucn3-Cre;Robo1*^*Δ/Δ*^,*2*^*flx/flx*^ mice with mice carrying a *Rosa26-Lox-Stop-Lox-H2BmCherry* reporter^62^ (Fig. 6A). In the resulting progeny, any cell that has ever expressed the *Insulin* promoter in the former or the *Ucn3* promoter in the latter is permanently marked with nuclear histone H2B-mCherry expression. The results obtained using this lineage tracing system showed no significant transdifferentiation of β cells to other cell types in the islet core in either *Ucn3-Cre;Robo1*^*Δ/Δ*^,*2*^*flx/flx*^ mice or *Ins2-Cre;Robo1*^*Δ/Δ*^,*2*^*flx/flx*^ mice (Fig. 6B, Supplemental Fig. 5). We thus concluded that the disrupted islet architecture in *Robo KO* lines and the increase in α cell ratio seen in the *Ucn3-Cre;Robo1*^*Δ/Δ*^,*2*^*flx/flx*^ mice are likely not caused by defects in β cell differentiation or maturation, accelerated β cell death, or transdifferentiation of β cells to α cells.

**Figure 7:**
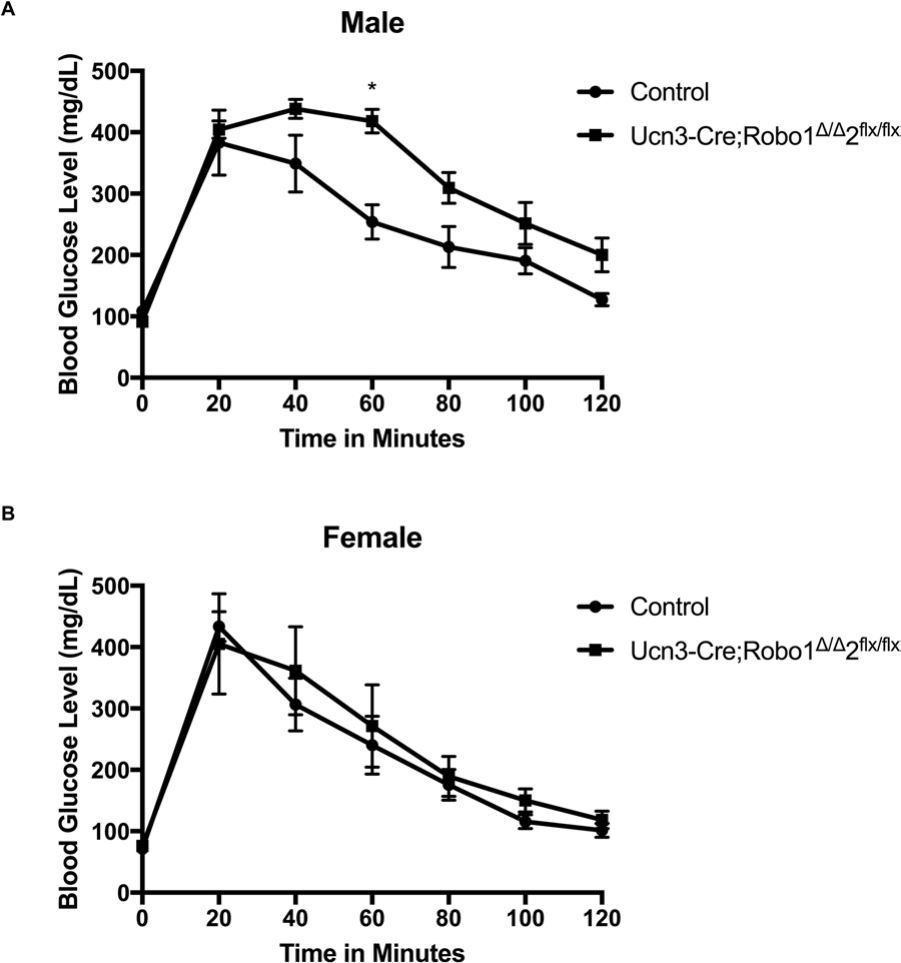
Robo receptors are important for glucose tolerance in male mice. **(A)** Glucose Tolerance Test on male control *Ucn3-Cre;Robo1*^+*/*+^,*2*^+*/*+^ and *Ucn3-Cre;Robo1*^*Δ/Δ*^,*2*^*flx/flx*^ mice fasted overnight showing mild glucose intolerance. **(B)** Glucose Tolerance Test on female control *Ucn3-Cre;Robo1*^+*/*+^,*2*^+*/*+^ and *Ucn3-Cre;Robo1*^*Δ/Δ*^,*2*^*flx/flx*^ mice fasted overnight showing no glucose intolerance.

Finally, we examined the glucose regulation of *Ucn3-Cre;Robo1*^*Δ/Δ*^,*2*^*flx/flx*^ mice. Isolation of islets for *in vitro* glucose stimulated insulin secretion (GSIS) is not feasible, because islets of *Robo KO* nice spontaneously dissociate upon isolation, likely due to cell adhesion defects. We thus performed *in vivo* intraperitoneal glucose tolerance tests (IPGTT) on *Ucn3-Cre;Robo1*^*Δ/Δ*^,*2*^*flx/flx*^ mice, compared to control colony-mates at the same age. Male *Ucn3-Cre;Robo1*^*Δ/Δ*^,*2*^*flx/flx*^ mice displayed slight glucose intolerance after overnight fasting. However, female *Ucn3-Cre;Robo1*^*Δ/Δ*^,*2*^*flx/flx*^ mice did not show glucose intolerance at the same conditions (Fig. 7).

## Discussion

In this study, we show that Robo receptors are essential for endocrine cell type sorting and mature architecture of the islets of Langerhans in mice. Conditional deletion of *Robo2* in the islets on the background of whole-body deletion of *Robo1* completely abolished the characteristic core-mantle organization of the islets. Instead of sorting to the periphery, α cells and δ cells became intermingled with β cells throughout the islet. The same phenotype was observed upon deletion of *Robo* in all endocrine cells, using a *Neurog3-Cre;Robo1*^*Δ/Δ*^,*2*^*flx/flx*^ model and upon selective deletion of *Robo* only in the β cell compartment, using the *Ins2-Cre;Robo1*^*Δ/Δ*^,*2*^*flx/flx*^ and *Ucn3-Cre;Robo1*^*Δ/Δ*^,*2*^*flx/flx*^ models. These results are in agreement with a recent study showing that α cells are dispensable for morphogenesis of the islets of Langerhans^63^, thus further highlighting the importance of β cells as the primary organizer of islet architecture. It should be noted that none of the three Cre lines used in this study are endocrine-specific. The *Ucn3* promotor is inherently expressed in some cell types in the central nervous system^64^, and the *Neurog3-Cre* and *Ins2-Cre* lines have been shown to have leaky expression in the brain and other neuroendocrine tissues^65,66^. While this can influence behavior and glucose regulation, it is unlikely that leakiness expression of the transgenes in different tissues accounts for the remarkably similar defects in endocrine cell type sorting and islet architecture in all three models.

We further show, using *Ucn3-Cre;Robo1*^*Δ/Δ*^,*2*^*flx/flx*^ mice, that deletion of *Robo* selectively in mature β cells, after initial islet formation has been completed, also results in loss of endocrine cell type sorting and improper islet architecture. One possible explanation for this observation is that Robo receptors are required for active maintenance of endocrine cell sorting. Ucn3 is a marker of functionally mature β cells in mice, which starts being expressed in β cells around P10^19,53^. While Ucn3-expressing β cells are functionally mature^52^, the islets of P13-P16 mice still undergo proliferative expansion^67^. Thus, a more plausible explanation to the phenotype observed in islets of *Ucn3-Cre;Robo1*^*Δ/Δ*^,*2*^*flx/flx*^ mice is that postnatal islets require Robo receptors to achieve mature islet architecture and endocrine cell type sorting during the expansion period. Future experiments, namely conditional deletion of Robo after weaning^68^, will resolve the question of active *versus* passive control of endocrine cell sorting and islet architecture in the adult. However, it would be interesting to see whether the observed reduction in Robo expression in islets from obese mice and type-2 diabetic humans accounts for the disruption of islet architecture during the islets’ compensatory expansion under these conditions.

One notable difference between prenatal and postnatal deletion of *Robo* is the effect on α-to-β cell ratio. While prenatal deletion of *Robo* did not result in a significant difference in α-to-β cell ratio, postnatal *Robo* mutants showed increased α cell proportions. Our lineage-tracing experiments, as well as the trend towards larger β cell area in the mutant, strongly suggest that the increase in α cells in the islets of *Ucn3-Cre;Robo1*^*Δ/Δ*^,*2*^*flx/flx*^ mice is not the result of β cells transdifferentiating into α cells. One possible explanation for the difference in α-to-β cell ratio observed between deletion of *Robo* prenatally using *Neurog3-cre* and *Ins2-Cre* and that observed when *Robo* is deleted postnatally with *Ucn3-Cre* is that the former two models delete *Robo* in all β cells, while the latter model only deletes the gene only in mature β cells, thus potentially having a differential influence on the neogenic niche of virgin β cells at the islet periphery^52^.

Defects in endocrine cell type sorting and islet architecture have previously been associated with defects in β cell maturation and/or differentiation^54–61^. Using mature β cell-selective lineage tracing, we found no difference in the proportion of label-carrying non-β cells between islets from *Robo KO* mice and controls, suggesting that deletion of Robo does not cause β cells to lose their differentiated identity. This conclusion is supported by the apparently normal expression of the maturation markers MafA and Ucn3 in β cells of *Robo KO* mice. Furthermore, the similar or larger total β cell area in *Robo KO* and control islets, the lack of increase in cell death in *Robo KO* islets, and the similar or larger overall islet size between islets of *Robo KO* models and their respective controls strongly suggest that the defects seen in endocrine cell type sorting and islet architecture are not due to loss of β cells.

Robo receptors have been implicated in collective cell movement not only in migrating axons, but also in other mammalian tissues^42,43^. They can function together with their canonical ligands, Slit1-3, or by partnering with other cell surface molecules to regulate cell adhesion and cell motility^27^. Based on results shown here, we propose that the defects in endocrine cell type sorting and islet architecture seen upon deletion of *Robo* in β cells are caused either by the inability of the cells to sense positional cues, the inability of the cells to rearrange their own cytoskeleton in response to positional cues, or the inability of the cells to attach to neighboring cells or to the extracellular matrix to facilitate movement.

Taken together, our data shown here demonstrate that expression of Robo receptors in β cells is required for endocrine cell type sorting and mature architecture in the islets of Langerhans. These findings raise the possibility of using Robo to prevent and reverse the loss of correct islet architecture in type-2 diabetics, as well as for directing the architecture of islet-like clusters derived by stem cell differentiation to make *bona fide* islets *in vitro* for transplantation into type-1 diabetics.

## Materials and Methods

### Animals

The experimental protocol for animal usage was reviewed and approved by the University of Wisconsin-Madison Institutional Animal Care and Use Committee (IACUC) under Protocol #M005221, and all animal experiments were conducted in accordance with the University of Wisconsin-Madison IACUC guidelines under the approved protocol. *Robo1*^*Δ*^,*2*^*flx*^^34^, *Ins2-Cre*^50^, *Neurog3-Cre*^46^, *Urocortin3-Cre*^52^ and *Rosa26-Lox-Stop-Lox-H2BmCherry*^62^ mice were previously described. All mouse strains were maintained on a mixed genetic background. Control colony mates in all analyses were either *Robo1*^+*/Δ*^,*2*^+*/flx*^, *Robo1*^+*/*+^,*2*^+*/*+^, or *Robo1*^+*/*+^,*2*^+*/*+^ with the corresponding *Cre* transgene (see Supplemental Fig. 1). The specificity of Cre expression and degree of conditional deletion of *Robo2* are shown in Supplemental Fig. 6.

### Immunofluorescence

Pancreata were fixed with 4% PFA at room temperature for 1 h, embedded in 30% sucrose and frozen in OCT (Tissue-Tek). Pancreatic sections (10 µm) were stained using a standard protocol. The following primary antibodies and dilutions were used: Guinea Pig anti-Insulin,1:800, (Dako), Rabbit anti-Glucagon, 1:200, (Cell Signaling), Goat anti-Somatostatin, 1:100, (Santa Cruz), Rabbit anti-Urocortin3, 1:500, (Phoenix) and Rabbit anti-MafA, 1:200, (Cell Signaling). The following secondary antibodies were used: Donkey anti-Guinea Pig 594 (Jackson), Donkey anti-Guinea Pig 647 (Jackson), Donkey anti-Rabbit 488 (Invitrogen), Donkey anti- Rabbit 594 (Invitrogen), Donkey anti-goat 647 (Invitrogen). TUNEL labeling was performed using the CF488A TUNEL Assay Apoptosis Detection Kit (Biotium). Slides were imaged using a Leica SP8 Scanning Confocal microscope or a Zeiss Axio Observer.Z1 microscope.

### Cell counting analysis

Cells were counted at maximum intensity projected images from 8–10, 10 µm thick confocal z-stacks using the ImageJ cell counter tool. Only cells with visible DAPI stained nuclei were counted. Cells were considered to be in the islet mantle if they fell within the 2 outermost cell layers of the islet, or core if they fell within any layers deeper than the outermost 2 cell layers. Only islets with at least 40 cells, with 10 or more cells being non β cells were counted for determining core/mantle localization.

### Islet size and shape analysis

Islet size and shape analyses were performed on the same images used for cell counting. All non-islet nuclei were erased using the ImageJ outline and clear outside functions. The subsequent images were then converted to 8 bit and set as threshold. Islet size and shape was determined using the Analyze Particles macro of ImageJ, which gives both a readout of area in µm^2^, and a Circularity Index that is calculated using the formula *circularity* = *4*π*(area/perimeter*^2^*)*. β cell area analysis was performed on four whole pancreas sections per mouse, that were at least 100 µm apart. Whole sections were stained for insulin and DAPI, and then imaged as 10x tile scans on a Leica SP8 Scanning Confocal microscope. Using ImageJ, each channel was converted to threshold, background was erased, and the area was measured using the Analyze Particles function. To obtain the β cell area per section, the insulin area was divided by the DAPI area for each section and converted to a percentage.

### Statistical analysis

Data are presented as average ± SEM unless otherwise indicated. All *P* values were calculated using a Student’s *t*-test using Prism 7 Graph Pad. *P* values of less than 0.05 were considered significant.

### Data availability

All data generated during and/or analyzed during the current study are available from the corresponding author on reasonable request.

## Electronic supplementary material

Supplemental Materials
