# Supplementary material for: Endocrine cell type sorting and mature architecture in the islets of Langerhans require expression of Roundabout receptors in β cells
Source: Sci Rep. 2018 Jul 18;8:10876. doi: 10.1038/s41598-018-29118-x (PMC6052079; doi:10.1038/s41598-018-29118-x)
Supplement: Supplementary file 1 — Supplemental Materials [file 41598_2018_29118_MOESM1_ESM.pdf]

# Supplementary Information

**Title: Endocrine cell type sorting and mature architecture in the islets of Langerhans require expression of Roundabout receptors in β cells**

Melissa T. Adams[1], Jennifer M. Gilbert[1], Jesus Hinojosa Paiz[1], Faith M. Bowman[1], Barak Blum[1]*

[1]Department of Cell and Regenerative Biology, University of Wisconsin-Madison School of Medicine and Public Health, 1111 Highland Ave., Madison, WI 53705

*Corresponding author. Email: bblum4@wisc.edu

**Supplemental Figure 1:**

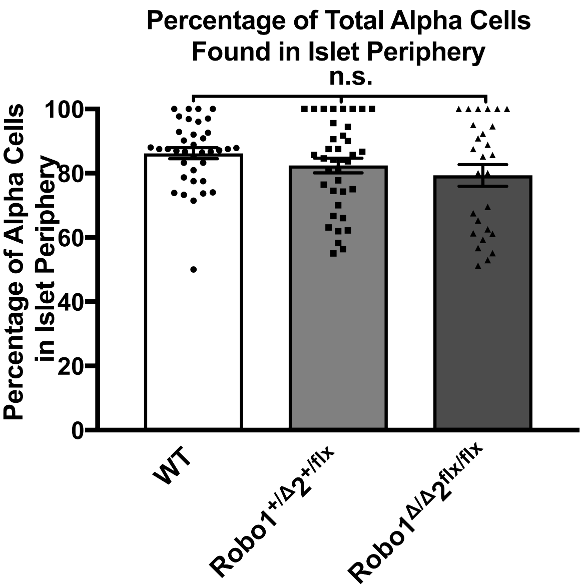

**Supplemental Figure 2:**

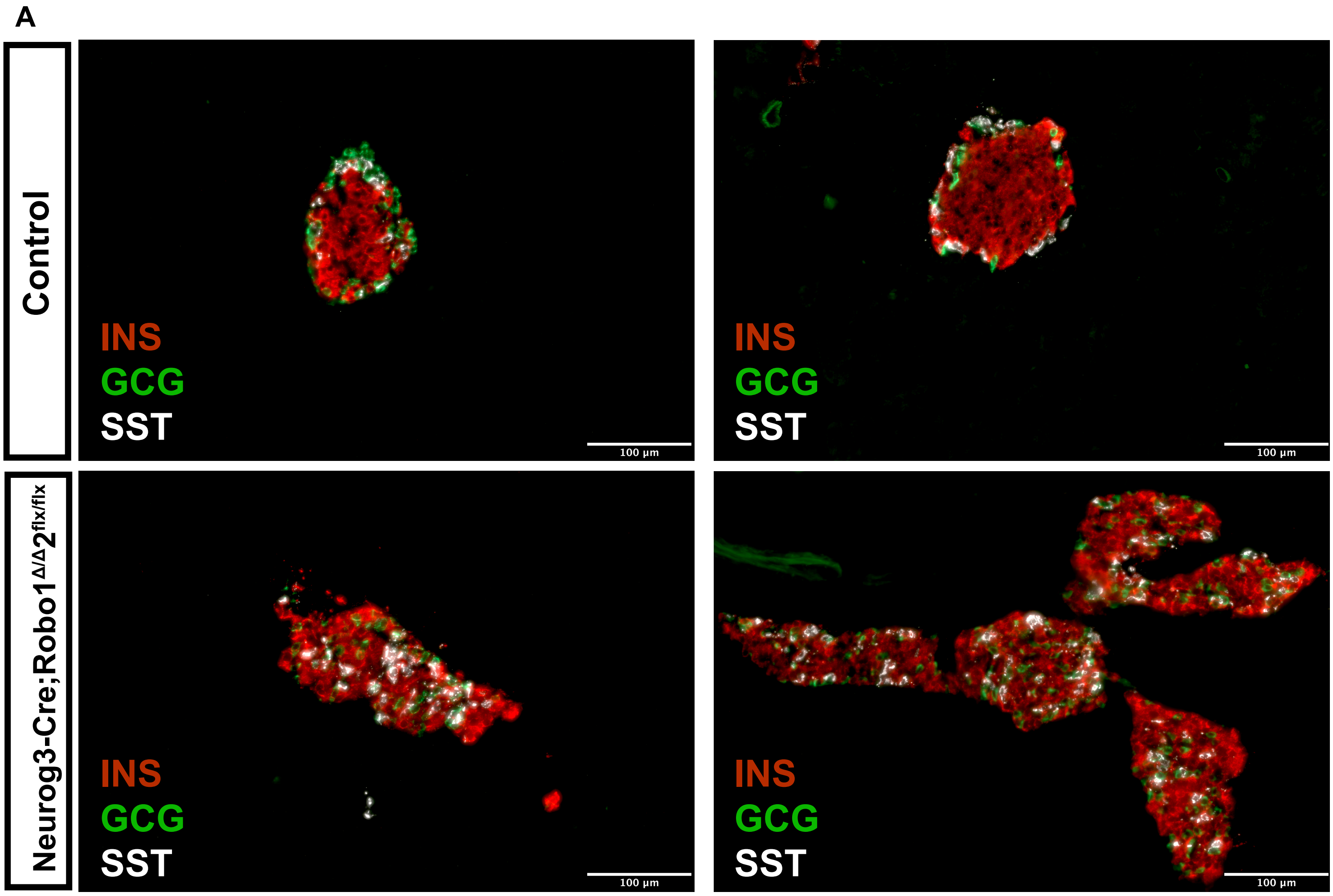


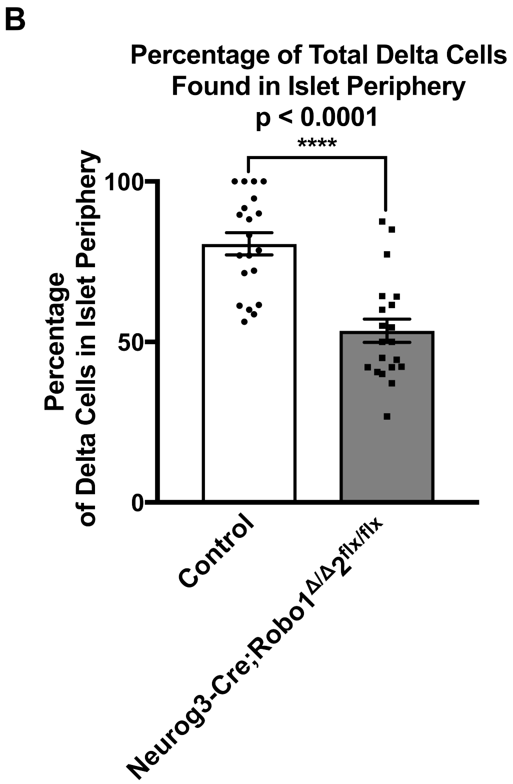

**Supplemental Figure 3:**

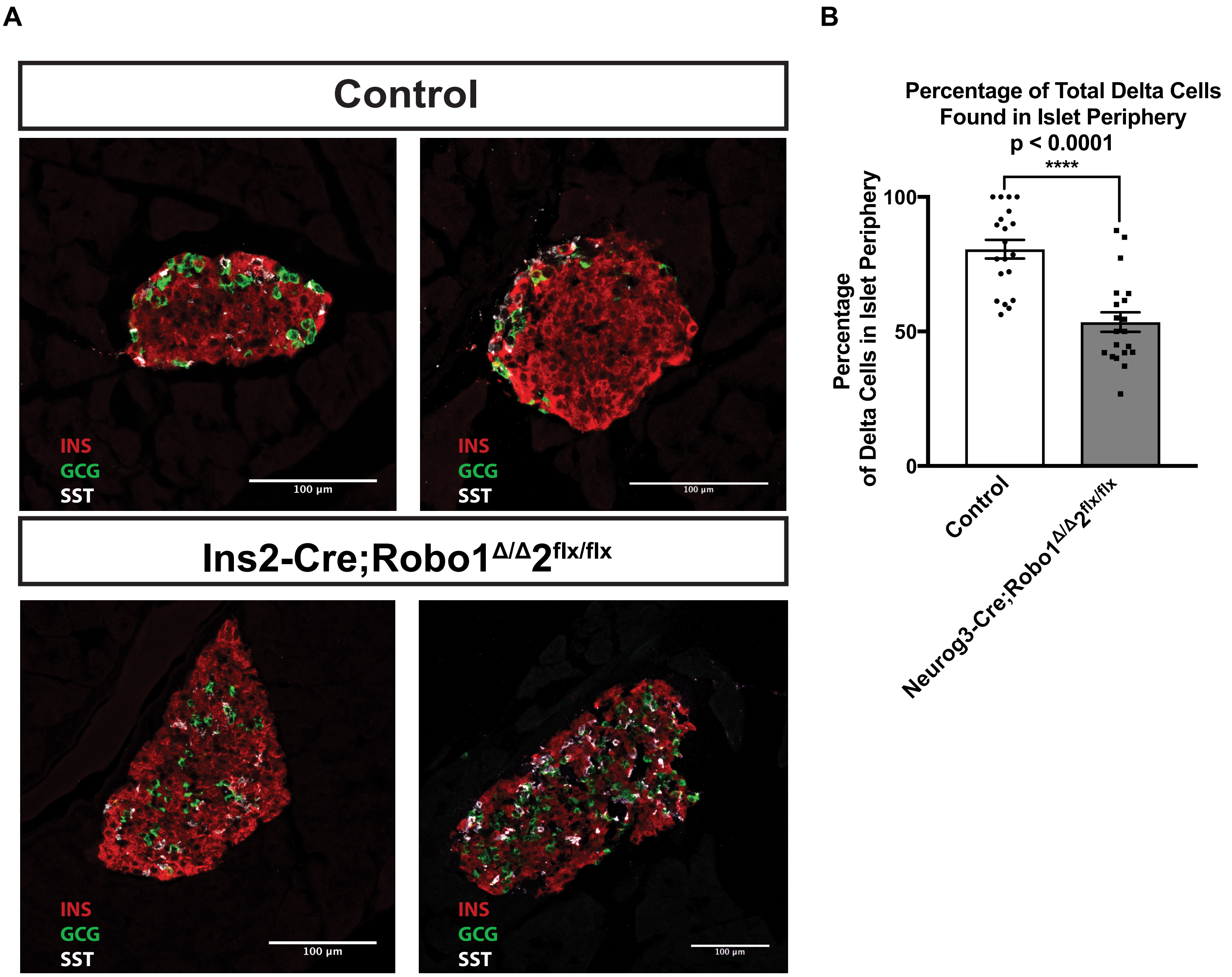

**Supplemental Figure 4:**

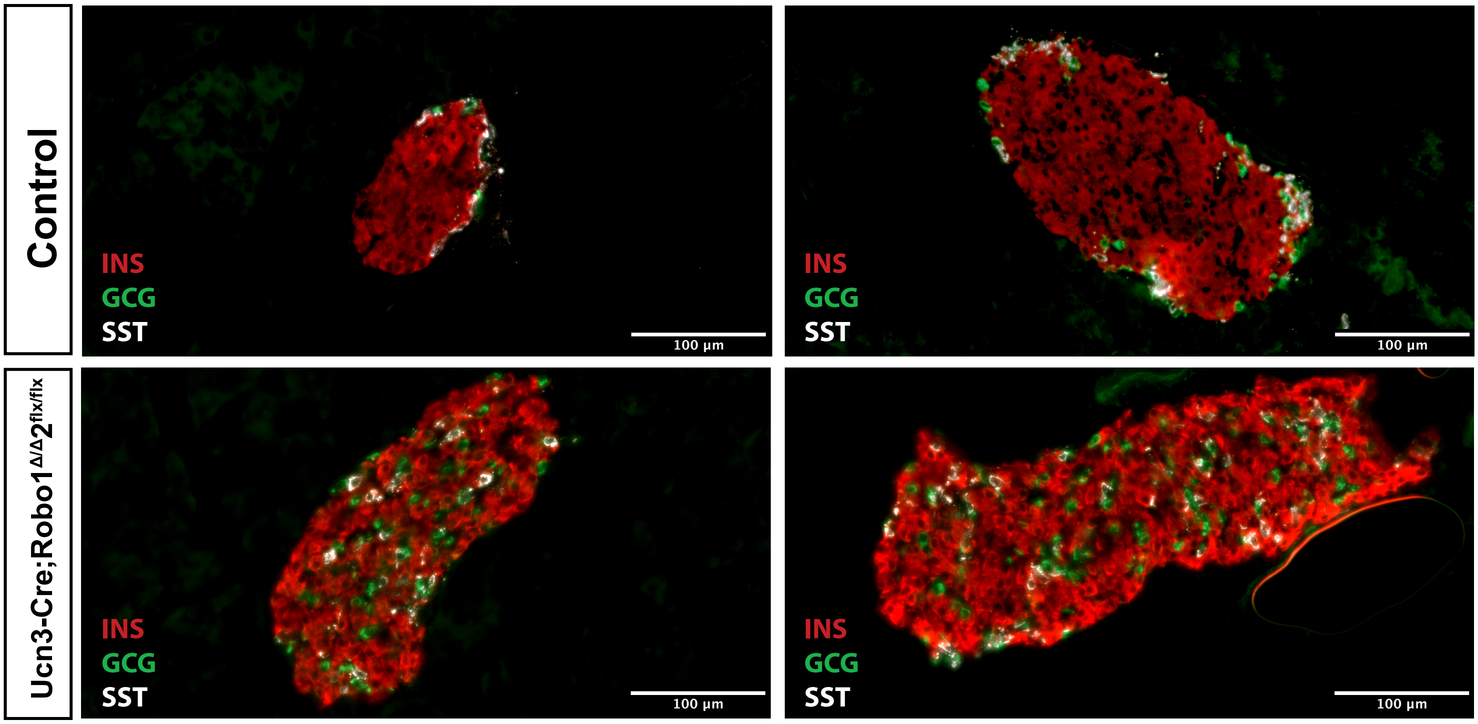

**Supplemental Figure 5:**

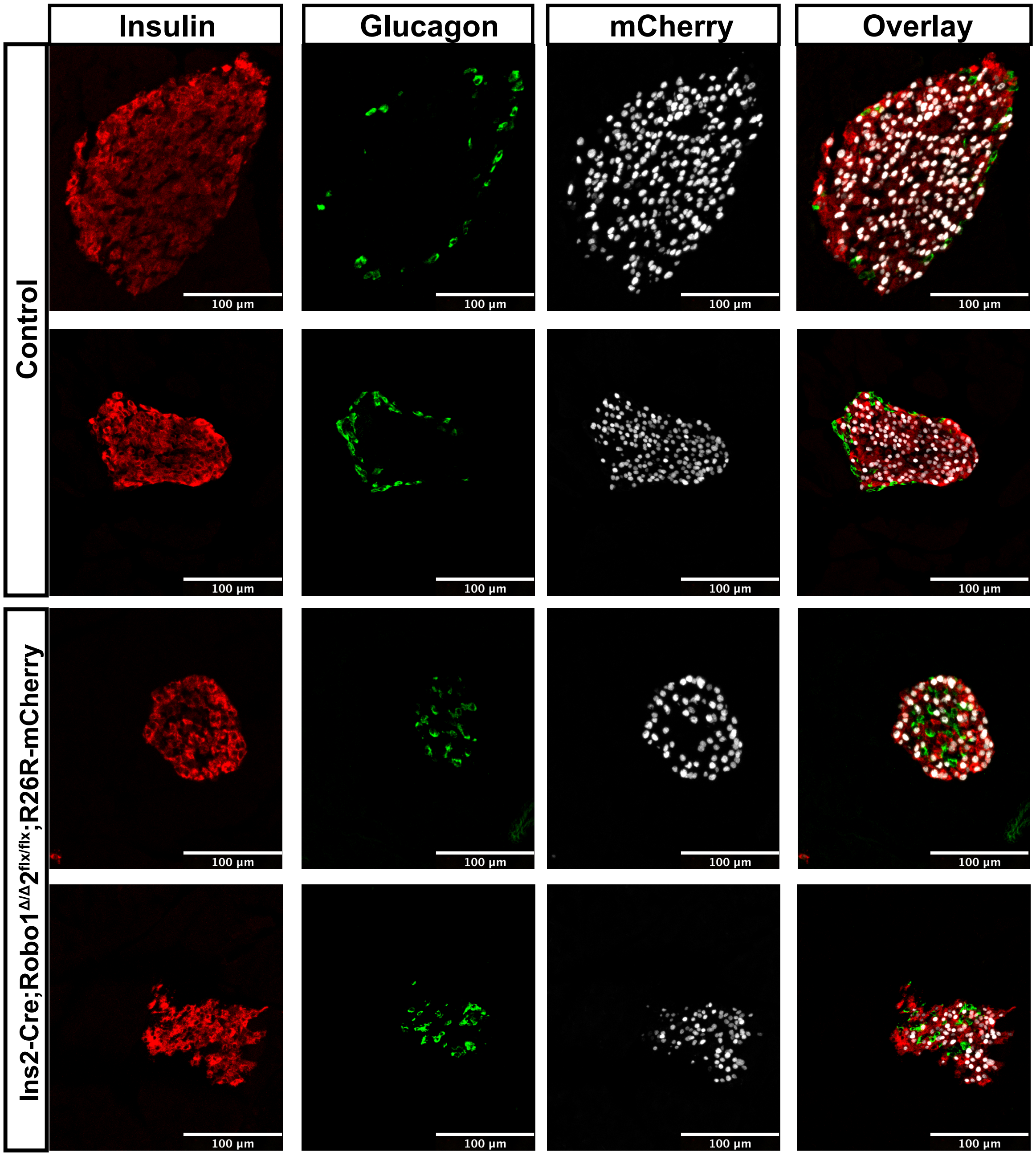

**Supplemental Figure 6:**

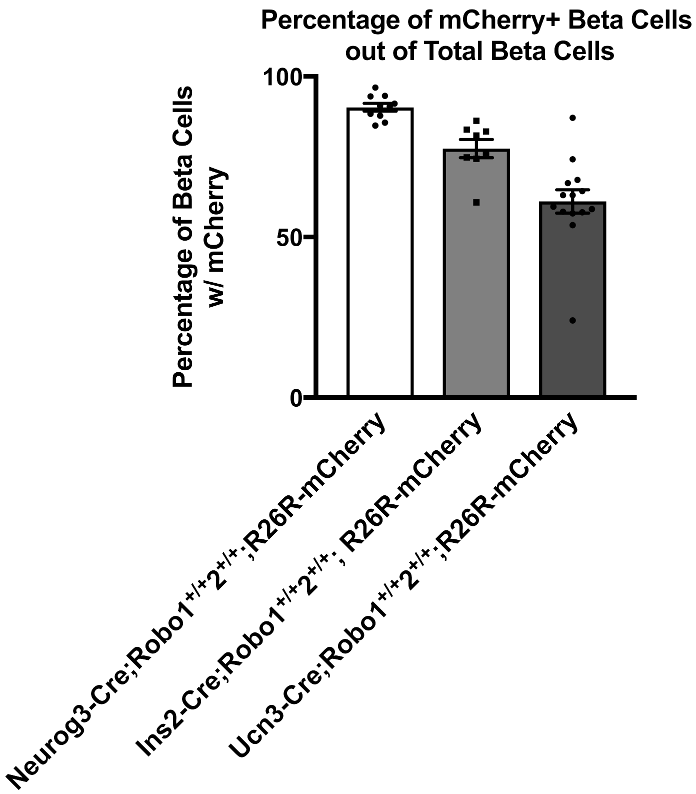

**Supplemental Figure 7:**

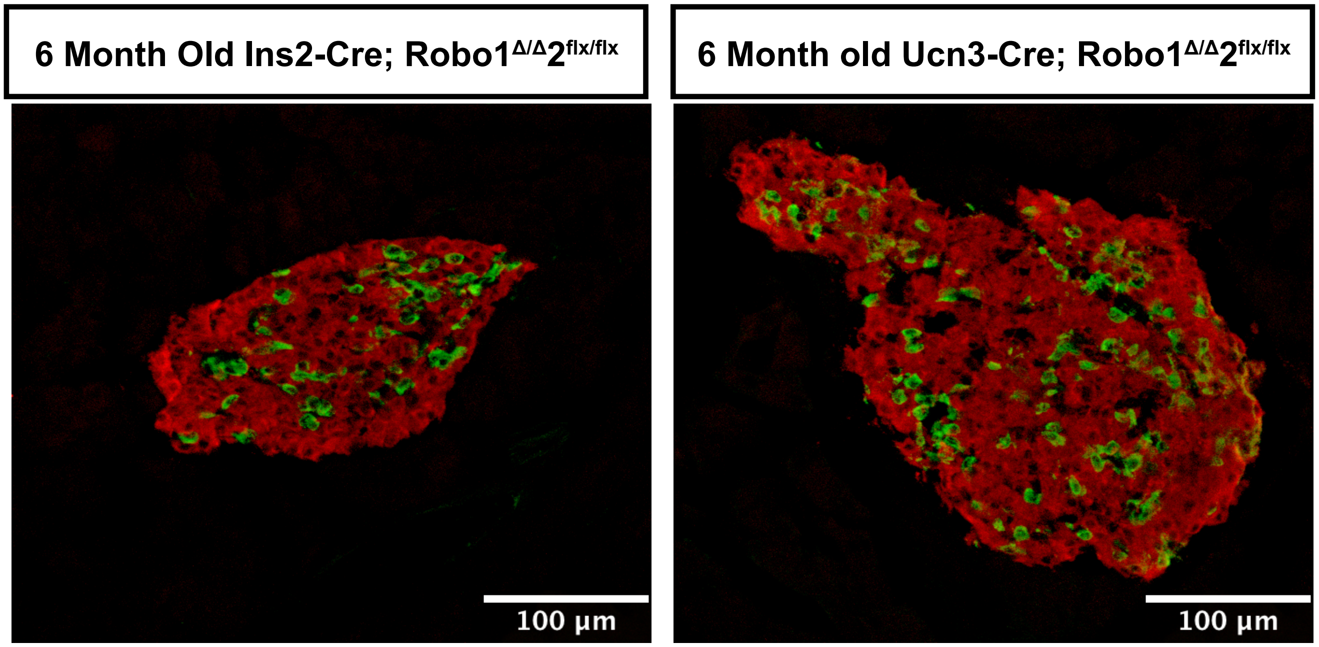

**Supplemental Figure 1.** Percentage of total α cells found in periphery of islet in WT, *Robo1*$^{+/\Delta}$*2*$^{+/flx}$, and *Robo1*$^{\Delta/\Delta}$*2*$^{flx/flx}$ mice showing no difference in islet architecture.

**Supplemental Figure 2. (A)** Immunofluorescent staining for Glucagon (green) showing α cells, Insulin (red) showing β cells, and Somatostatin (white) showing δ cells of control (*Robo1*$^{+/\Delta}$*2*$^{+/flx}$ and *Neurog3-Cre; Robo1*$^{+/+}$*2*$^{+/+}$) and *Neurog3-Cre; Robo1*$^{\Delta/\Delta}$*2*$^{flx/flx}$ islets from 2 month old mice. **(B)** Percentage of total δ cells found in periphery in control (*Robo1*$^{+/\Delta}$*2*$^{+/flx}$ and *Neurog3-Cre; Robo1*$^{+/+}$*2*$^{+/+}$) *vs*. *Neurog3-Cre; Robo1*$^{\Delta/\Delta}$*2*$^{flx/flx}$ islets.

**Supplemental Figure 3. (A)** Immunofluorescent staining for Glucagon (green) showing α cells, Insulin (red) showing β cells, and Somatostatin (white) showing δ cells of control (*Robo1*$^{+/+}$*2*$^{+/+}$ and Ins2-*Cre; Robo1*$^{+/+}$*2*$^{+/+}$) and *Ins2-Cre; Robo1*$^{\Delta/\Delta}$*2*$^{flx/flx}$ islets from 2 month old mice. **(B)** Percentage of total δ cells found in periphery in control (*Robo1*$^{+/+}$*2*$^{+/+\ +}$ and *Ins2-Cre; Robo1*$^{+/+}$*2*$^{+/+}$) vs. *Ins2-Cre; Robo1*$^{\Delta/\Delta}$*2*$^{flx/flx}$ islets.

**Supplemental Figure 4.** Immunofluorescent staining for glucagon (green) showing α cells, Insulin (red) showing β cells, and somatostatin (white) showing δ cells of control (*Robo1*$^{+/+}$*2*$^{+/+}$ and *Ucn3-Cre; Robo1*$^{+/+}$*2*$^{+/+}$) and *Ucn3-Cre; Robo1*$^{\Delta/\Delta}$*2*$^{flx/flx}$ islets from 1.5-3 month old mice.

**Supplemental Figure 5.** Control *Ins2-Cre; Robo1*$^{+/+}$*2*$^{+/+}$ and *Ins2-Cre; Robo1*$^{\Delta/\Delta}$*2*$^{flx/flx}$ islets with lineage traced β cells expressing nuclear mCherry (white), counterstained for Insulin (red), Glucagon (green), and DAPI (blue), showing no transdifferentiation of α to β cells.

**Supplemental Figure 6.** Quantification of mCherry positive β cells out of total β cells per islet in *Neurog3-Cre; Robo1*$^{+/+}$*2*$^{+/+}$, *Ins2-Cre; Robo1*$^{+/+}$*2*$^{+/+}$, and *Ucn3-Cre; Robo1*$^{+/+}$*2*$^{+/+}$ islets all with lineage traced β cells expressing nuclear mCherry from the *R26R-mCherry* construct.

**Supplemental Figure 7.** Immunofluorescence staining for β cells (Insulin, red) and α cells (glucagon, green) of Ins2-*Cre; Robo1*$^{\Delta/\Delta}$2$^{flx/flx}$ and *Ucn3-Cre; Robo1*$^{\Delta/\Delta}$2$^{flx/flx}$, islets from 6 month old mice showing disrupted islet architecture.
